# Sustained Immunogenicity of 2-dose Human Papillomavirus 16/18 AS04-adjuvanted Vaccine Schedules in Girls Aged 9–14 Years: A Randomized Trial

**DOI:** 10.1093/infdis/jix154

**Published:** 2017-06-07

**Authors:** Li-Min Huang, Thanyawee Puthanakit, Chiu Cheng-Hsun, Tang Ren-Bin, Tino Schwarz, Angelo Pellegrino, Susanna Esposito, Louise Frenette, Shelly McNeil, Paolo Durando, Paul Rheault, Carlo Giaquinto, Michael Horn, Karl Ulrich Petry, Klaus Peters, Toma Azhar, Peter Hillemanns, Stephanie De Simoni, Damien Friel, Suryakiran Pemmaraju, Marjan Hezareh, Florence Thomas, Dominique Descamps, Nicolas Folschweiller, Frank Struyf

**Affiliations:** 1 Department of Pediatrics, National Taiwan University Children’s Hospital, National Taiwan University, Taipei,; 2 Department of Pediatrics, Chang Gung Children’s Hospital, Chang Gung University, Taoyuan, and; 3 Department of Pediatrics, Cheng Hsin General Hospital, Taipei, Taiwan;; 4 Division of Infectious Diseases, Department of Pediatrics, Faculty of Medicine, and Research Unit in Pediatric Infectious Diseases and Vaccines, Chulalongkorn University, Thailand;; 5 Central Laboratory and Vaccination Centre, Klinikum Würzburg Mitte, Standort Juliusspital, Würzburg,; 6 Pediatric Office Dr. Med. Michael Horn, Schoenau am Koenigssee,; 7 Department of Gynaecology and Obstetrics, Klinikum Wolfsburg, Wolfsburg,; 8 Facharzt für Frauenheilkunde und Geburtshilfe, Hamburg, and; 9 Medizinische Hochschule Hannover, Hannover, Germany;; 10 Department Distretto di Dronero, Azienda Sanitaria Locale Cuneo 1, Cuneo,; 11 Pediatric Highly Intensive Care Unit, Department of Pathophysiology and Transplantation, Università degli Studi di Milano, Fondazione IRCCS Ca’ GrandaOspedale Maggiore Policlinico, Milan,; 12 Department of Health Sciences, University of Genoa and IRCCS AOU San Martino-IST, Genoa, and; 13 Department of Women’s and Children’s Health, University of Padova, Padua, Italy;; 14 Q&T Research Incorporated, Sherbrooke,; 15 Canadian Center for Vaccinology, IWK Health Centre and Capital Health, Dalhousie University, Halifax,; 16 Medicor Research Inc, Sudbury, and; 17 Manna Research, Toronto, Canada;; 18 GSK, and; 19 Chiltern International for GSK, Wavre, Belgium; and; 20 GSK, Bangalore, India

**Keywords:** human papillomavirus (HPV), 2-dose schedule, cervical cancer, Cervarix.

## Abstract

**Background.:**

We previously reported the noninferiority 1 month after the last dose of 2-dose human papillomavirus 16/18 AS04-adjuvanted (AS04-HPV-16/18) vaccine schedules at months 0 and 6 (2D_M0,6) and months 0 and 12 (2D_M0,12) in girls aged 9–14 years compared with a 3-dose schedule at months 0, 1, and 6 (3D_M0,1,6) in women aged 15–25 years. Here, we report the results at study end (month 36 [M36]).

**Methods.:**

Girls were randomized 1:1 and received 2 vaccine doses either 6 months (2D_M0,6) or 12 months apart (2D_M0,12); women received 3 doses at months 0, 1, and 6 (3D_M0,1,6). Endpoints included noninferiority of HPV-16/18 antibodies for 2D_M0,6 versus 3D_M0,1,6; 2D_M0,12 versus 3D_M0,1,6; and 2D_M0,12 versus 2D_M0,6; and assessment of neutralizing antibodies, T cells, B cells, and safety.

**Results.:**

At M36, the 2D_M0,6 and 2D_M0,12 schedules remained noninferior to the 3D_M0,1,6 schedule in terms of seroconversion rates and 3D/2D geometric mean titers for anti-HPV-16 and anti-HPV-18. All schedules elicited sustained immune responses up to M36.

**Conclusions.:**

Both 2-dose schedules in young girls remained noninferior to the 3-dose schedule in women up to study conclusion at M36. The AS04-HPV-16/18 vaccine administered as a 2-dose schedule was immunogenic and well tolerated in young girls.

## Summary

Human papillomavirus vaccination began using a 3-dose schedule. Compared with the 3-dose schedule in women, the 2-dose schedules are effective options in young girls as of 36 months after first dose. Two-dose schedules could improve compliance and vaccination coverage.

Cervical cancer is one of the most common cancers among women worldwide. Approximately 528000 new cervical cancer cases and 266000 deaths occurred in 2012 worldwide [1, 2]. Most human papillomavirus (HPV) infections clear naturally, but persistent infections lead to cervical cancer. Human papillomavirus 16 and HPV-18 are the most common among high-risk HPV types and are responsible for approximately 70% of cervical cancer cases [3–7]. Universal mass HPV vaccination began approximately 10 years ago using a 3-dose schedule, and prophylactic HPV vaccination programs with the AS04-HPV-16/18 vaccine (Cervarix; GSK) and the 4-valent HPV vaccine (Gardasil; Merck & Co) have been shown to contribute to the reduction in HPV prevalence [8–13].

The initially licensed schedule for the AS04-HPV-16/18 vaccine is comprised of 3 doses administered at months 0, 1, and 6 (3D_M0,1,6). However, high vaccine coverage and compliance rates can be difficult to achieve with the 3-dose regimen. The high immune response to the vaccine observed in the younger population led to the investigation of a 2-dose schedule [14].

The current phase III confirmatory study evaluated the immunogenicity and safety of the AS04-HPV-16/18 vaccine administered as 2 alternative 2-dose schedules: a 2-dose schedule at months 0 and 6 (2D_M0,6) or a 2-dose schedule at months 0 and 12 (2D_M0,12) in girls aged 9–14 years compared with the standard 3D_M0,1,6 schedule in women aged 15–25 years. We previously reported that both 2-dose schedules were immunologically noninferior to the standard 3D_M0,1,6 schedule up to 13 months after the first dose.[15] Because long-term protection following HPV vaccination is important, subjects were followed through 36 months after vaccination. Here, we report study results up to 36 months after the first vaccine dose.

## METHODOLOGY

### Study Design and Participants

This study was a phase IIIb, multicenter, open-label, randomized trial (ClinicalTrials.gov NCT01381575) conducted in 5 countries (Canada, Germany, Italy, Taiwan, and Thailand) between 2011 and 2014 [15, 16]. Healthy girls aged 9–14 years were randomized (1:1) to either the 2D_M0,6 group or the 2D_M0,12 group. Women aged 15–25 years who received the 3D_M0,1,6 schedule served as the control group. The inclusion/exclusion criteria, the study population, randomization and masking, and vaccine composition were previously described [15].

The primary objective to demonstrate the noninferiority of the 2D_M0,6 schedule as compared with the 3D_M0,1,6 schedule at month 7 was met and described previously [15]. 

Secondary objectives included evaluation of the noninferiority of the reduced schedules as compared with the 3D_M0,1,6 schedule until study conclusion at month 36 (M36), assessment of anti–HPV-16/18 neutralizing antibodies (nAbs), HPV-16/18–specific T-cell and memory B-cell responses, and safety.

The study protocol, all amendments, and informed consent were reviewed and approved by an independent ethics committee or institutional review board. The study was designed and conducted according to the principles from the Declaration of Helsinki, Good Clinical Practice guidelines and all other applicable regulatory requirements. Written informed consent was obtained from every participant and/or the parent/legally authorized representative.

### Immunogenicity Assessments

Anti–HPV-16/18 antibodies were assessed in all participants by enzyme-linked immunosorbent assay (ELISA), with the assay cutoff of 8 ELISA units (EU)/mL for anti–HPV-16 and 7 EU/mL for anti–HPV-18 up to month 12. The assay cutoff was revised from month 18 onward to increase precision [15]; hence 19 EU/mL for anti–HPV-16 and 18 EU/mL for anti–HPV-18 were used from month 18 to month 36.[15] Human papillomavirus 16/18 nAbs, T cells, and B cells were determined in a subset of participants, as previously described [17]. Human papillomavirus 16/18 nAbs were determined by pseudovirion-based neutralization assay (PBNA) with a cutoff of 40 estimated dose 50% (ED_50_; serum dilution giving a 50% reduction of the signal compared with a control without serum). CD4+ and CD8+ T cells specific to HPV-16/18/31/45 were evaluated by intracellular cytokine staining. B-cell responses to HPV-16/18/31/45 were assessed by B-cell enzyme-linked immunosorbent spot assay.

### Safety Assessments

The occurrence of serious adverse events (SAEs); the occurrence of SAEs related to the investigational product, to study participation, to GSK concomitant products or any fatal SAE; the occurrence of medically significant conditions; and the occurrence of pregnancy and pregnancy outcomes were recorded throughout the study period in all groups.

### Statistical Methods

The primary analyses of immunogenicity were based on the M36 according-to-protocol (ATP) cohort for immunogenicity (ATP-I); secondary immunogenicity analyses based on the M36 total vaccinated cohort (TVC) were performed to complement the ATP analyses. Safety analyses were based on the TVC.

The ATP-I at M36 included all participants who met all of the eligibility criteria, complied with the study procedures, and for whom data concerning immunogenicity endpoint measures were available. The TVC at M36 included all participants who received at least 1 dose of the study vaccine and for whom data were available at the follow-up visit. Noninferiority and descriptive immunogenicity analyses were based on the initially seronegative participants in the ATP-I cohort.

Seroconversion was defined as the appearance of antibodies (ie, titer greater than or equal to the cutoff value) in the serum of subjects seronegative before vaccination. Seropositivity was defined as an antibody titer greater than or equal to the cutoff value. The geometric mean titer (GMT) calculations were performed by taking the antilog of the mean of the log titer transformations. Antibody titers below the cutoff of the assay were given an arbitrary value of half the cutoff for the purpose of GMT calculation. Seroconversion and seropositivity rates for each antigen and GMTs were calculated with exact 95% confidence intervals (CIs) before and after vaccination.

The frequencies of HPV-16/18/31/45–specific CD4^+^ T cells producing cytokines and memory B cells were summarized.

To assess the noninferiority, the following between-group comparisons were performed. For noninferiority in terms of seroconversion rates, for each HPV antigen, the upper limit (UL) of the 2-sided standardized asymptotic 95% confidence interval of the difference between the percentages of seroconverted subjects in the 3-dose schedule and the 2-dose schedule (or between the two 2-dose schedules) was computed. If noninferiority in terms of seroconversion rates was reached (UL of the 2-sided standardized asymptotic 95% confidence interval of the difference in the percentage of seroconverted subjects in both groups was <5%), the 2-sided 95% confidence intervals of GMT ratios were computed using an analysis of variance (ANOVA) model on the log10 transformation of the titers at each timepoint. Noninferiority in terms of GMTs was demonstrated if the UL of 95% confidence interval for the GMT ratio for both groups was <2. The ANOVA model included the vaccine group as fixed effect.

Noninferiority was assessed sequentially first for the 2D_M0,6 versus 3D_M0,1,6, and then for 2D_M0,12 versus 3D_M0,1,6 and 2D_M0,12 versus 2D_M0,6. Noninferiority was tested at all timepoints, and if not reached, analysis at the subsequent timepoint was not performed.

The sample size was calculated to provide sufficient power to compare the 2D_M0,6 group with the 3D_M0,1,6 group up to the study end (M36). A total of 1428 subjects allowed for the detection of a 5% difference between the 2D_M0,6 and the 3D_M0,1,6 groups for seroconversion rates and a 2-fold difference for GMTs with 91% power at study conclusion (M36).

## RESULTS

### Study Population

**Figure 1.**
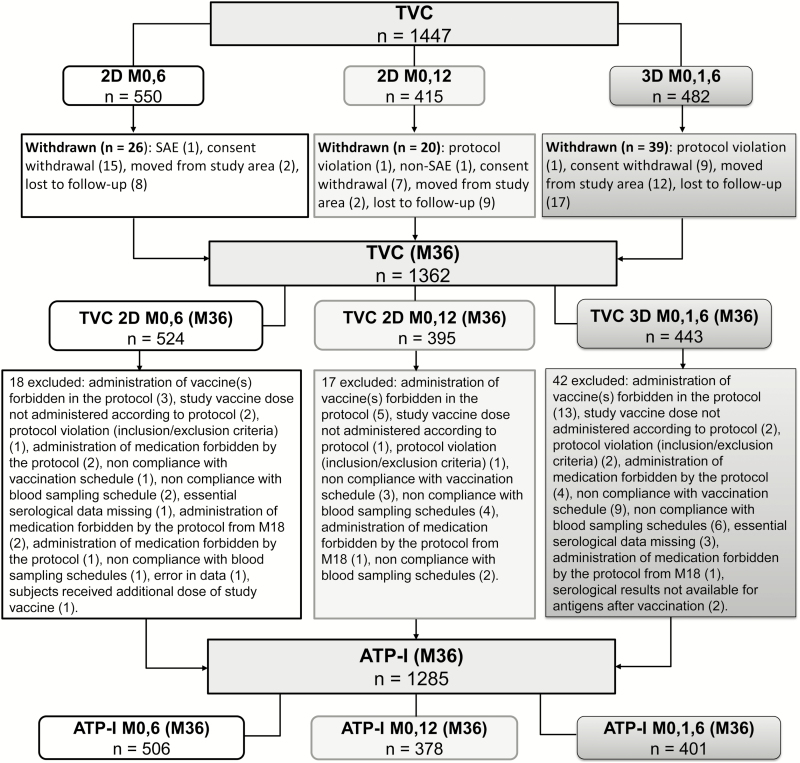
Flow of participants. Abbreviations: 2D_M0,6, 2-dose schedule administered at months 0 and 6; 2D_M0,12, 2-dose schedule administered at months 0 and 12; 3D_M0,1,6, 3-dose schedule administered at months 0, 1, and 6; ATP-I, according-to-protocol cohort for immunogenicity; M, month; SAE, serious adverse event; TVC, total vaccinated cohort.

A total of 1447 participants were included in the study; 1362 of 1447 (94.1%) completed the M36 visit. A total of 1285 of 1362 (88.8%) participants were included in the ATP-I cohort at M36 (Figure 1). The first subject was enrolled in the study on 29 June 2011, and the last study visit for M36 was on 13 November 2014. This report presents data from the follow-up visits at M36.

Due to the temporary unavailability of the allocated vaccine for the 2D_M0,12 group at some study sites, the randomization system allocated girls to the 2D_M0,6 group at a higher rate. Although this led to an imbalance in terms of the number of participants in both 2-dose groups, no impact on the validity of the study was noted because a sufficient number of girls were randomized to the 2D_M0,12 group to allow evaluation of study objectives (Table 1). Demographic characteristics and baseline HPV serostatus of the study participants in the ATP-I are available in the Supplementary Materials.

**Table 1.** Demographic Characteristics and Baseline Human Papillomavirus Serostatus of the Study Participants in the Total Vaccinated Cohort

	2D_M0,6 girls aged 9–14 y	2D_M0,12 girls aged 9–14 y	3D_M0,1,6 women aged 15–25 y
Patient Characteristics	n = 524	n = 395	n = 443
Age, y, at time of first vaccine dose, mean (SD)	14.5 (1.6)	14.3 (1.6)	22.5 (3.1)
Geographic ancestry, no. (%)			
African heritage / African American	4 (0.8)	6 (1.5)	3 (0.7)
Asian—Central/South Asian heritage	1 (0.2)	2 (0.5)	1 (0.2)
Asian—East Asian heritage	139 (26.5)	71 (18.0)	104 (23.5)
Asian—South East Asian geritage	105 (20.0)	103 (26.1)	103 (23.3)
White—Arabic / North African heritage	1 (0.2)	1 (0.3)	0 (0.0)
White—Caucasian / European heritage	269 (51.3)	210 (53.2)	229 (51.7)
Other	5 (1.0)	2 (0.5)	3 (0.7)
HPV-16 baseline serostatus, no. (%)			
Seronegative^a^	468 (89.3)	353 (89.4)	365 (82.4)
Seropositive	54 (10.3)	42 (10.6)	76 (17.2)
Not available	2 (0.4)	0 (0.0)	2 (0.4)
HPV-18 baseline serostatus, no. (%)			
Seronegative^a^	476 (90.8)	371 (93.9)	392 (88.5)
Seropositive	42 (8.0)	21 (5.3)	49 (11.0)
Not available	6 (1.2)	3 (0.8)	2 (0.5)

Abbreviations: 2D_M0,6, 2-dose schedule administered at months 0 and 6; 2D_M0,12, 2-dose schedule administered at months 0 and 12; 3D_M0,1,6, three-dose schedule administered at months 0, 1, and 6; HPV, human papillomavirus; SD, standard deviation; TVC, total vaccinated cohort.

^a^Seronegative status defined as an antibody titer lower than the assay cutoff before vaccination (19 enzyme-linked immunosorbent assay units [EU]/mL for HPV-16 and 18 EU/mL for HPV-18).

### Immunogenicity

#### Noninferiority Assessment

The primary objective of the study was to demonstrate the immunological noninferiority of the AS04-HPV-16/18 vaccine when administered according to the 2D_M0,6 schedule as compared with the 3D_M0,1,6 vaccination schedule at 1 month after last dose. This objective was previously reported [12].

At M36, in the ATP-I, the 2D_M0,6 schedule remained noninferior to the 3D_M0,1,6 schedule in terms of seroconversion rates and GMT ratios for both antigens (Table 2). Noninferiority was also shown for all intermediate timepoints (data not shown).

**Table 2.** Noninferiority Assessment of Human Papillomavirus 16 and Human Papillomavirus 18 Antibody Responses in Initially Seronegative Participants at Month 36 (According-to-Protocol Cohort for Immunogenicity)

Antibody	Group	Age, y	No.	Seroconversion, % (95% CI)	GMT, EU/mL (95% CI)	Seroconversion difference^a^, % (95% CI)	GMT ratio^b^, (95% CI)
2D_M0,6 vs 3D_M0,1,6						3D − 2D_M0,6	3D/2D_M0,6
HPV-16	2D_M0,6	9–14	455	100 (99.2–100)	1210.2 (1124.8–1302.1)	0.00 (−1.15 to 0.84)	1.10 (0.97–1.24)
	3D_M0,1,6	15–25	330	100 (99.0–100)	1326.4 (1193.9–1473.5)	…	…
HPV-18	2D_M0,6	9-14	462	99.8 (98.8–100)	562.8 (516.4–613.4)	−0.06 (−1.37 to 0.96)	0.98 (0.85–1.13)
	3D_M0,1,6	15–25	356	99.8 (98.4–100)	552.6 (494.1–618.0)	…	…
2D_M0,12 vs 3D_M0,1,6						3D − 2D_M0,12	3D/2D_M0,12
HPV-16	2D_M0,12	9–14	339	100 (98.9–100)	1559.3 (1431.2–1699.0)	0.00 (−1.15 to 1.12)	0.85 (0.74–0.97)
	3D_M0,1,6	15–25	330	100 (98.9–100)	1326.4 (1193.9–1473.5)	…	…
HPV-18	2D_M0,12	9–14	355	100 (99.0–100)	804.0 (731.8–883.4)	−0.28 (−1.58 to 0.79)	0.69 (0.59–0.8)
	3D_M0,1,6	15–25	356	99.7 (98.4–100)	552.6 (494.1–618.0)	…	…
2D_M0,12 vs 2D_M0,6						2D_M0,6 − 2D_M0,12	2D_M0,6/2D_M0,12
HPV-16	2D_M0,12	9–14	339	100 (98.9–100)	1559.3 (1431.2–1699.0)	0.00 (−0.84 to 1.12)	0.78 (0.69–0.87)
	2D_M0,6	9–14	455	100 (99.2–100)	1210.2 (1124.8–1302.1)	…	…
HPV-18	2D_M0,12	9–14	355	100 (99.0–100)	804.0 (731.8–883.4)	−0.22 (−1.22 to 0.86)	0.70 (0.62–0.80)
	2D_M0,6	9–14	462	99.8 (99.8–100)	562.8 (516.4–613.4)	…	…

Seronegative status was defined as an antibody titer lower than the assay cutoff before vaccination (assay cutoff of 8 enzyme-linked immunosorbent assay units [EU]/mL for anti–human papillomavirus [HPV] 16 and 7 EU/mL for anti–HPV-18 up to month 12, 19 EU/mL for anti–HPV-16 and 18 EU/ml for anti–HPV-18 from month 18 to month 36).

Two-sided 95% confidence intervals of geometric mean antibody titer ratios between groups were computed using an analysis of variance on log10 transformed titers, including vaccine group as a fixed effect.

Abbreviations: 2D_M0,6, 2-dose schedule administered at months 0 and 6; 2D_M0,12, 2-dose schedule administered at months 0 and 12; 3D_M0,1,6, 3-dose schedule administered at months 0, 1, and 6; CI, confidence interval; ELISA, enzyme-linked immunosorbent assay; EU, enzyme-linked immunosorbent assay units; GMT, geometric mean antibody titer; HPV, human papillomavirus.

^a^Noninferiority was demonstrated if the upper limit of the 95% confidence interval for difference in seroconversion rates was less than the predefined limit of 5%.

^b^Noninferiority was demonstrated if the upper limit of the 95% confidence interval for the geometric mean titer ratio was less than the predefined limit of 2.

At M36, noninferiority of the 2D_M0,12 compared with the 2D_M0,6 and the 3D_M0,1,6 schedules was demonstrated as well (Table 2).

#### Immune Responses to Human Papillomavirus Vaccine Types 16 and 18

At M36, in each group, all initially seronegative participants in the ATP-I were seropositive for HPV-16 antibodies, and all but 2 (1 in the 2D_M0,6 group and 1 in the 3D_M0,1,6 group) were seropositive for HPV-18 antibodies.

**Figure 2.**
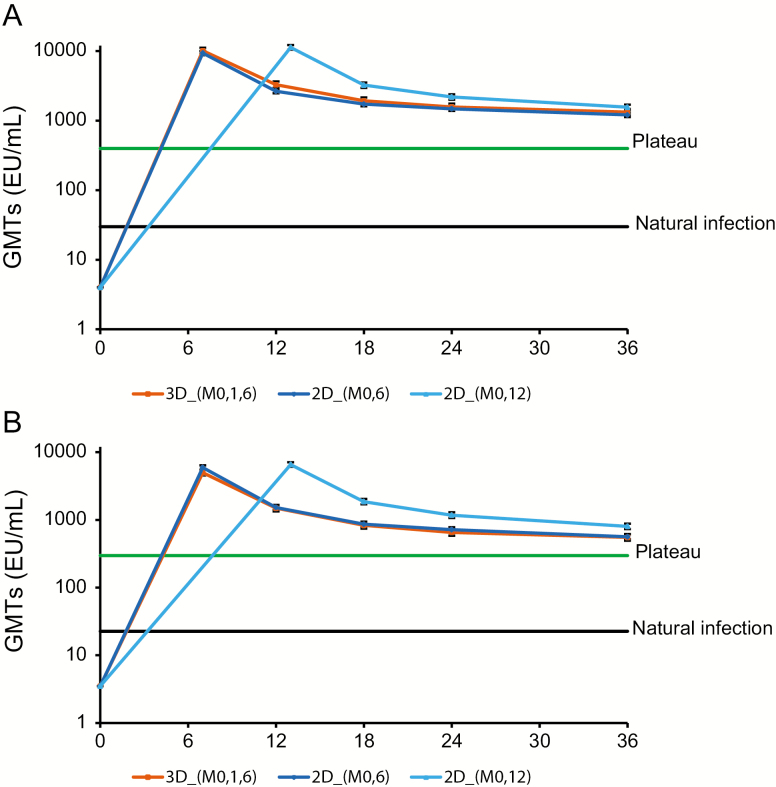
Anti–human papillomavirus (HPV) 16 (*A*) and anti–HPV-18 (*B*) antibody levels in initially seronegative participants at month 36 (according-to-protocol cohort for immunogenicity). Natural infection: Geometric mean titers in women aged 15–25 years who had cleared a natural infection in a previous trial (NCT00122681). Geometric mean titers were 29.8 and 22.7 enzyme-linked immunosorbent assay units (EU)/mL for HPV-16 and HPV-18 antibodies, respectively [34]. Plateau: Geometric mean titers at the plateau level (month 45–50 timepoint) in women aged 15–25 years who received 3 doses of the HPV-16/18 AS04–adjuvanted vaccine at months 0, 1, and 6 in a previous trial (NCT00120848). Geometric mean titers were 397.8 and 297.3 EU/mL for HPV-16 and HPV-18 antibodies, respectively [22]. The error bars represent 95% confidence intervals. Abbreviations: 2D_M0,6, 2-dose schedule administered at months 0 and 6 (n = 339); 2D_M0,12, 2-dose schedule administered at months 0 and 12 (n = 330); 3D_M0,1,6, 3-dose schedule administered at months 0, 1, and 6 (n = 455); EU, enzyme-linked immunosorbent assay units; GMTs, geometric mean titers.

After a peak response at month 7, GMTs for anti–HPV-16/18 antibodies gradually declined until month 18, and reached a plateau between month 18 and M36. In initially seronegative participants, at M36, GMTs for anti–HPV-16 and anti–HPV-18 antibodies were 1210.2 EU/mL (95% CI = 1124.8–1302.1) and 562.8 EU/mL (95% CI = 516.4–613.4) in the 2D_M0,6 group; 1559.3 EU/mL (95% CI = 1431.2–1699.0) and 804.0 EU/mL (95% CI = 731.8–883.4) in the 2D_M0,12 group; and 1326.4 EU/mL (95% CI = 1193.9–1473.5) and 552.6 EU/mL (95% CI = 494.1–618.0) in the 3D_M0,1,6 group, respectively (Figure 2).

**Figure 3.**
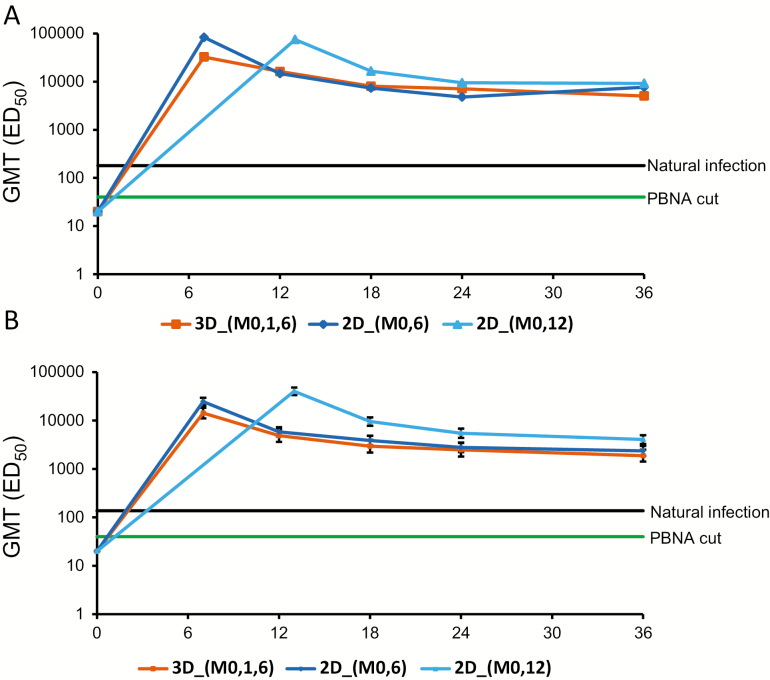
Anti–human papillomavirus (HPV) 16 (*A*) and anti–HPV-18 (B) neutralizing antibody levels in initially seronegative participants at month 36 (according-to-protocol cohort for immunogenicity). Pseudovirion-based neutralization assay (PBNA) cutoff: 40 ED_50_. Natural infection: Geometric mean titers corresponding to natural infection in a previous study. Geometric mean titers measured by PBNA were 180.1 ED_50_ for HPV-16 and 137.3 ED_50_ for HPV-18 [35]. The error bars represent 95% confidence intervals. Abbreviations: 2D_M0,6, 2-dose schedule administered at months 0 and 6 (n = 88); 2D_M0,12, 2-dose schedule administered at months 0 and 12 (n = 85); 3D_M0,1,6, 3-dose schedule administered at months 0, 1, and 6 (n = 95); ED_50_, estimated dose: serum dilution giving a 50% reduction of the signal compared to a control without serum; GMT, geometric mean titer; PBNA, pseudovirion-based neutralization assay.

At M36, all of the initially seronegative subjects in all 3 groups had seroconverted for both anti–HPV-16 and anti–HPV-18 nAbs when measured by PBNA (Figure 3). For the 2D_M0,6 group, GMTs at M36 were 7660.2 ED_50_ (95% CI = 6131.7–9569.7) for anti–HPV-16 nAbs and 2365.5 ED_50_ (95% CI = 1868.2–2995.2) for anti–HPV-18 nAbs; for the 2D_M0,12 group, GMTs were 9214.3 ED_50_ (95% CI = 7112.3–11937.5) for anti–HPV-16 nAbs and 4046.4 ED_50_ (95% CI = 3278.0–4994.8) for anti–HPV-18 nAbs; for the 3D_M0,1,6 group, GMTs were 5035.0 ED_50_ (95% CI = 3726.9–6802.0) for anti–HPV-16 nAbs and 1881.4 ED_50_ (95% CI = 1417.7–2496.7) for anti–HPV-18 nAbs.

**Figure 4.**
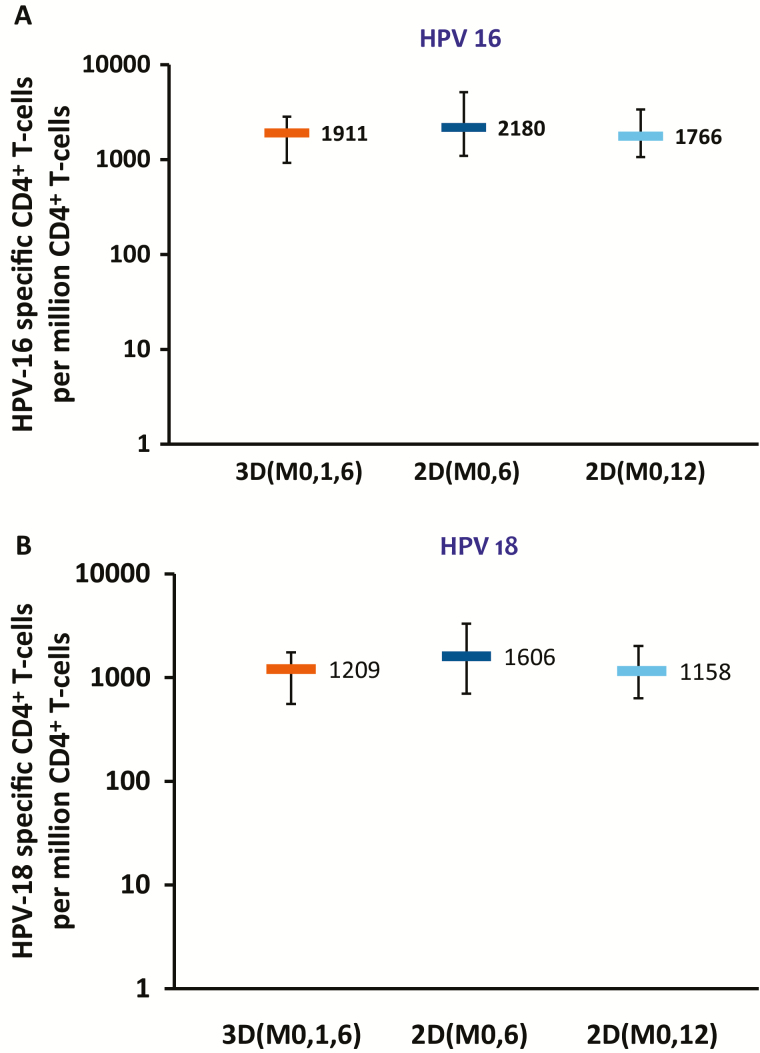
Human papillomavirus (HPV) 16–specific (*A*) and HPV-18–specific (*B*) CD4^+^ T-cell responses at month 36 (according-to-protocol cohort for immunogenicity). Boxplots show median, lower quartile, and upper quartile frequency of cells. Abbreviations: 2D_M0,6, 2-dose schedule administered at months 0 and 6 (n = 75 [*A*] and 72 [*B*]); 2D_M0,12, 2-dose schedule administered at months 0 and 12 (n = 70 [*A*] and 75 [*B*]); 3D_M0,1,6, 3-dose schedule administered at months 0, 1, and 6 (n = 57 [*A*] and 56 [*B*]); HPV, human papillomavirus.

**Figure 5.**
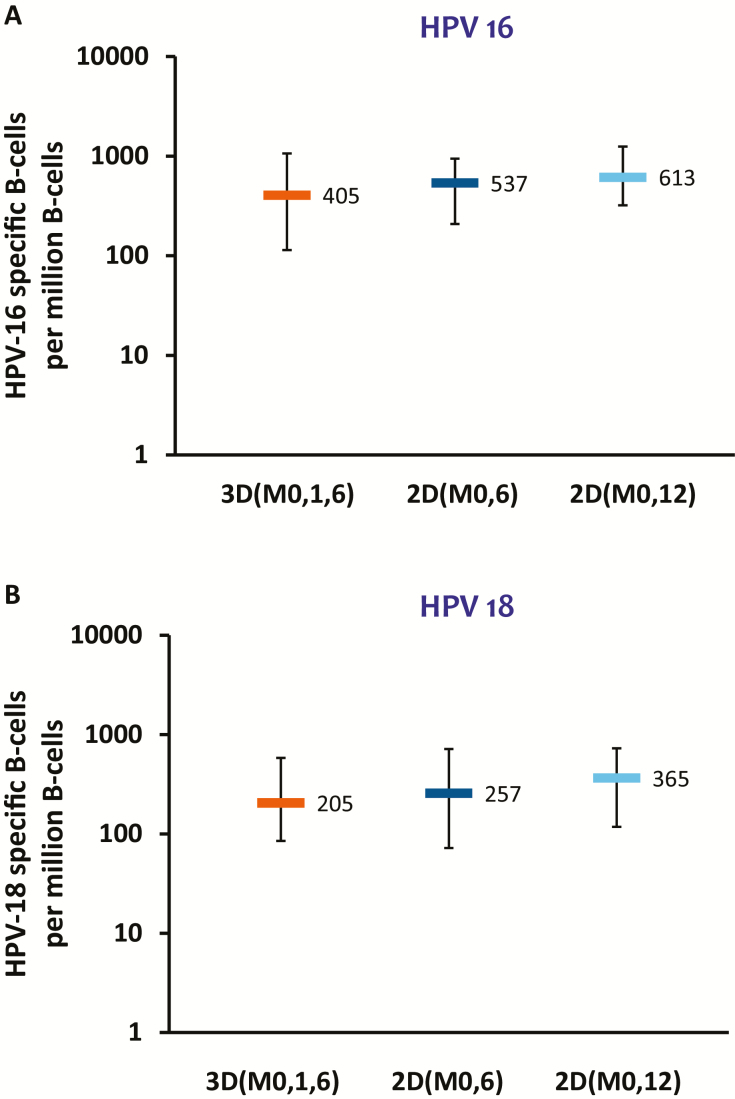
Human papillomavirus (HPV) 16–specific (*A*) and HPV-18–specific (*B*) B-cell responses at month 36 (according-to-protocol cohort for immunogenicity). Boxplots show median, lower quartile, and upper quartile frequency of cells. Abbreviations: 2D_M0,6, 2-dose schedule administered at months 0 and 6 (n = 70 [*A*] and 67 [*B*]); 2D_M0,12, 2-dose schedule administered at months 0 and 12 (n = 67 [*A*] and 72 [*B*]); 3D_M0,1,6, 3-dose schedule administered at months 0, 1 and 6 (n = 53 [*A*] and 54 [*B*]); HPV, human papillomavirus.

At M36, the frequencies of HPV-16/18–specific CD4^+^ T cells and memory B cells were within similar ranges for all groups (Figures 4 and 5). Measurable CD4^+^ T-cell responses were detected in the 2D_M0,6, 2D_M0,12, and 3D_M0,1,6 groups for HPV-31 and HPV-45. No substantial HPV-16/18/31/45–specific CD8^+^ T-cell responses were detected at any timepoint. At M36, median frequencies of HPV-31–specific B cells were 68.0 (2D_M0,6 group), 101.0 (2D_M0,12 group) and 27.0 (3D_M0,1,6 group), and median frequencies of HPV-45–specific Bcells were 59.0, 56.0, and 68.0 in these groups, respectively.

### Safety

Safety results included events that occurred since the study started, except reactogenicity following vaccination and potential immune-mediated diseases, which were previously published [15]. Up to M36, a total of 72 participants (n = 20 in the 2D_M0,6 group, n = 24 in the 2D_M0,12 group, and n = 28 in the 3D_M0,1,6 group) reported at least 1 SAE, none of which were fatal. One case of systemic lupus erythematosus was reported by 1 subject 264 days after the first dose in the 2D_0,12M group and was considered causally related to vaccination by the investigator. The subject only received 1 dose of vaccine, and the event was not resolved at end of study. None of the other SAEs were considered to be causally related to vaccination by the investigator. There was 1 withdrawal due to a nonserious adverse event in the 2D_M0,12 group at month 12 (the subject was diagnosed with celiac disease). There were no additional withdrawals due to SAEs during the course of the study. There was 1 withdrawal due to a nonvaccine-related SAE: immunoglobulin A–mediated nephritis.

Until the last follow-up visit at M36, a total of 374 participants (n = 134 in the 2D_M0,6 group, n = 87 in the 2D_M0,12 group, and n = 153 in the 3D_M0,1,6 group) reported at least 1 medically significant condition. A total of 36 pregnancies occurred during the entire study (n = 1 in each 2-dose group, and n = 34 in the 3D_M0,1,6 group); of those, 32 pregnancies resulted in live infants with no apparent congenital anomaly. One ectopic pregnancy, 2 elective terminations, and 1 stillbirth were recorded in the 3D_M0,1,6 group; none of these were considered by the investigator to be related to the vaccination.

## DISCUSSION

We previously demonstrated that the AS04-HPV-16/18 vaccine administered as a 2-dose schedule (either 6 months or 12 months apart) to girls aged 9–14 years elicited an immune response that was noninferior to the one elicited by 3 doses in women aged 15–25 years up to 6 months after the first dose.[15] These results led to the licensure of the 2-dose schedules for the AS04-HPV-16/18 vaccine, and subsequently, the World Health Organization updated its recommendation with the 2-dose schedule for girls aged <15 years [18]. In the current follow-up, we demonstrated that this observation was sustained until at least 36 months after the first dose. Noninferiority of a 2-dose HPV vaccination schedule compared with the standard 3-dose regimen has been reported in previous studies with the AS04-HPV-16/18 or the 4-valent HPV vaccine [14, 17, 19–23]. In a recent study, Iversen and colleagues showed the noninferiority of 2-dose schedules in girls aged 9–14 years versus the 3-dose regimen with the 9-valent vaccine (Merck & Co) 4 weeks after the last injection [24]. However, to our knowledge, this study is the first large-scale, phase III, multicountry trial of HPV vaccines that assessed the immunogenicity of 2 different 2-dose schedules (2D_M0,6 and 2D_M0,12) and the persistence up to 36 months after the first dose. The noninferiority of the 2D_M0,12 schedule versus not only the 3D_M0,1,6 schedule and but also the 2D_M0,6 schedule demonstrated at the end of this study confirms that flexibility around the administration of the second dose can be considered for 2-dose vaccination.

Due to ethical and practical reasons, efficacy studies on HPV vaccination cannot be conducted in young girls; hence this study was not designed to assess efficacy endpoints such as high-grade cervical intraepithelial neoplasia (CIN) or persistent HPV infection. In this study, the AS04-HPV-16/18 vaccine immunogenicity profiles were noninferior for the 2-dose schedules in girls compared with the 3-dose schedule in women aged 15–25 years, the age group in which the vaccine was shown to be efficacious against HPV-16/18–associated infections and high-grade cervical intraepithelial neoplasia in previous efficacy trials [25–30]. Protection against HPV infection being thought to be mainly antibody-mediated, it is likely that the 2-dose schedule of the AS04-HPV-16/18 vaccine administered to adolescent girls would result in a similar level of protection to that previously observed in young women who received the 3-dose schedule. A recent analysis of pooled efficacy data from the Costa Rica Vaccine Trial (CVT) and the Papilloma Trial Against Cancer in Young Adults (PATRICIA) suggested that 2 doses of the AS04-HPV-16/18 vaccine induced a similar protection against cervical HPV-16/18 infections as the 3-dose schedule, although most of the women received the vaccine only at month 0 and month 1, which is probably not optimal [31].

The vaccine safety profile was similar between the 3 groups, SAEs were rarely reported (5.1%), and the overall safety profile was consistent with results of the previous pooled analyses of HPV-16/18 AS04–adjuvanted vaccine clinical trials [32, 33]. One case of systemic lupus erythematosus was reported by a subject in the 2D_M0,12 group 264 days after the only dose received. The investigator considered that there was a reasonable possibility the systemic lupus erythematosus may have been caused by the vaccine, although no rationale was provided for the assessment and the systemic lupus erythematosus diagnosis could not be confirmed based on the information provided. Also, as previously described, a nonserious potential immune-mediated disease (VIIth nerve paralysis) reported in the 3-dose group was considered to have a possible causal relationship to vaccination and resolved 13 days after the first vaccination [15, 16]. 

A potential limitation of this study is the fact that no efficacy endpoints were assessed due to the age of participants (young girls). In addition, this study did not evaluate the 2-dose schedule in females aged >15 years.

In conclusion, the immunogenicity of the HPV-16/18 AS04–adjuvanted vaccine administered according to the 2-dose schedules at 6- or 12-month intervals to girls aged 9–14 years remained noninferior to the standard 3-dose schedule administered to young women aged 15–25 years at 36 months after the first administered dose. These results support the use of the 2-dose schedules for HPV vaccination in adolescent girls. Availability of both of these 2-dose schedules makes it more convenient for the subject, the prescriber, and mass vaccination campaigns.

## Supplementary Data

Supplementary materials are available at *The Journal of Infectious Diseases* online. Consisting of data provided by the authors to benefit the reader, the posted materials are not copyedited and are the sole responsibility of the authors, so questions or comments should be addressed to the corresponding author.

## Supplementary Material

Huang_HPV2D-schedules_Supplemental-material

## Notes

***Acknowledgments***. L.-M. H., C.-H. C., T. F. S., S. E., C. G., P. D., P. V. S., M. Hezareh, D. D., F. T., P. K., and F. S. participated in study design. T. P., L.-M. H., C.-H. C., R.-B. T., T. F. S., S. E., L. F., C. G., S. M., P. D., M. Horn, S. D. S., D. F., T. A., A. P., K. U. P., K. P., P. H., and P. V. S. acquired the data. L.-M. H., C.-H. C., R.-B. T., T. F. S., K. U. P., and S. E. extracted the data. S. E., P. V. S., and F. T. performed the analysis. T. P., L.-M. H., C.-H. C., T. F. S., S. E., C. G., S. M., P. D., S. D. S., D. F., B. D. M., P. V. S., D. D., F. T., K.P., K. U. P., and F. S. interpreted data. T. P., L.-M. H., C.-H. C., R.-B. T., T. F. S., S. E., L. F., C. G., S. M., P. R., P. D., T. A., A. P., K. U. P., K. P., P. H., and M. Horn provided study material or subject V. S. and F. T. participated in statistical analysis. L. F., S. D. S., and D. F. did the laboratory testing. L.-M. H., C.-H. C., R.-B. T., T. F. S., S. E., L. F., C. G., S. M., P. D., D. D., F. T., T. A., K. P., and F. S. supervised the study group. All authors had full access to the complete final study reports, reviewed the manuscript draft(s), and gave final approval to submit for publication.The authors thank all study participants and their families and all clinical-study site personnel who contributed to the conduct of this trial. Medical writing was provided by John Bean (John Bean Medical Writing, Halle, Belgium). The authors would like to thank Business & Decision Life Sciences platform for editorial assistance and manuscript coordination, on behalf of GSK. Thibaud André coordinated manuscript development and editorial support.

***Financial support.*** GlaxoSmithKline Biologicals SA funded the studies and was involved in all stages of study conduct, including analysis of the data. GlaxoSmithKline Biologicals SA also covered all costs associated with the development and publication of this article.

***Potential conflicts of interest.*** T. P. received a grant through their respective institution from the GSK group of companies. R. T. received funding from the GSK group of companies through his institution. L.-M. H. received grants through his institution from the GSK group of companies and also received consultancy fees for participation in the HPV expert board and payment for educational presentation from the GSK group of companies. T. F. S. received fees for board membership, consultancy, and payment for lectures, including service on speakers bureaus, from the GSK group of companies. S. E. received grants from the GSK group of companies, Crucell, Novartis, Pfizer, and Roche through her institution; payment for lectures, including service on speakers bureaus, from the GSK group of companies, Crucell, Novartis, and Astrazeneca; and support for travel to meetings for the study from the GSK group of companies. L. F. received support for travel to meetings for the study from the GSK group of companies. C. G. received payments for board membership and lectures, including service on speakers bureaus, from Sanofi Pasteur MSD, Merck, and the GSK group of companies. S. M. received grants through her institution from the GSK group of companies, Pfizer, and Sanofi Pasteur MSD; consultancy fees from Pfizer; and payment for lectures, including service on speakers bureaus, from Merck and Pfizer. P. R. received funding through his institution for the conduct of the clinical trial, received support for travel to meetings for the study from the GSK group of companies, and holds stock option from the GSK group of companies. P. D. received a grant from the GSK group of companies through his institution for the conduct of this trial; received grants through his institution from Sanofi Pasteur MSD, Berna Crucell, Novartis, and Pfizer for the conduct of other clinical trials; received support for travel to meetings from the GSK group of companies; and received consultancy fees for participation to advisory boards and payment for lectures, including service on speakers bureaus, from Pfizer and Sanofi Pasteur MSD. M. Horn received a grant from the GSK group of companies for the conduct of this study, consultancy fees from the GSK group of companies and Novartis, support for travel to meetings for the study from the GSK group of companies, payment for board membership from Novartis, and payment for lectures, including service on speakers bureaus, development of educational presentations, and travel, accommodation, and meeting expenses from the GSK group of companies, Sanofi Pasteur MSD, and Novartis. U. K. P. received a grant from the GSK group of companies through his institution for the conduct of this trial; received other grants through his institution from the GSK group of companies and Sanofi Pasteur MSD for the conduct of other clinical trials; and received personal fees from the GSK group of companies and Sanofi Pasteur MSD. A. T. received grants from the GSK group of companies through his institution for the conduct of this and other trials. P. H. received a grant from Medizinische Hochschule Hannover, Germany. S. D. S., D. F., P. S., F. T.s, D. D., N. F., and F. S. are employees of the GSK group of companies. M. Hezareh is a Chiltern International consultant for the GSK group of companies. F. T. holds stock options from the GSK group of companies. D. D., N. F., and F. S. hold restricted shares/shares in the GSK group of companies as part of their employee remuneration. All other authors: No reported conflicts. All authors have submitted the ICMJE Form for Disclosure of Potential Conflicts of Interest. Conflicts that the editors consider relevant to the content of the manuscript have been disclosed.
