# Supplementary material for: Sustained Immunogenicity of 2-dose Human Papillomavirus 16/18 AS04-adjuvanted Vaccine Schedules in Girls Aged 9–14 Years: A Randomized Trial
Source: J Infect Dis. 2017 Jun 7;215(11):1711–9. doi: 10.1093/infdis/jix154 (PMC5853959; doi:10.1093/infdis/jix154)
Supplement: Huang_HPV2D-schedules_Supplemental-material [file jix154_suppl_huang_hpv2d-schedules_supplemental-material.docx]

**Supplementary material**

**Supplementary Table 1. Demographic characteristics and baseline HPV serostatus of the study participants in the ATP-I**

|  | 2D_M0,6 Girls 9-14y | 2D_M0,12 Girls 9-14y | 3D_M0,1,6 Women 15-25y |
| --- | --- | --- | --- |
| ATP-I | N = 506 | N = 378 | N = 401 |
| Age (years) at time of first vaccine dose, mean (SD) | 14.5 (1.6) | 14.3 (1.6) | 22.4 (3.1) |
| Geographic ancestry  African Heritage / African American  American Indian or Alaskan Native  Asian - Central/South Asian Heritage  Asian - East Asian Heritage  Asian - Japanese Heritage  Asian - South East Asian Heritage  Native Hawaiian or Other Pacific Islander  White - Arabic / North African Heritage  White - Caucasian / European Heritage  Other | 4 (0.8)  0 (0.0)  1 (0.2)  136 (26.9)  0 (0.0)  104 (20.6)  0 (0.0)  1 (0.2)  255 (50.4)  5 (1.0) | 6 (1.6)  0 (0.0)  1 (0.3)  69 (18.3)  0 (0.0)  101 (26.7)  0 (0.0)  1 (0.3)  198 (52.4)  2 (0.5) | 3 (0.7)  0 (0.0)  1 (0.2)  99 (24.7)  0 (0.0)  95 (23.7)  0 (0.0)  0 (0.0)  200 (49.9)  3 (0.7) |
| HPV-16 baseline serostatus, n (%) |  |  |  |
| Seronegative | 455 (89.9) | 339 (89.7) | 330 (82.3) |
| Seropositive | 49 (9.7) | 39 (10.3) | 69 (17.2) |
| Not available | 2 (0.4) | 0 (0.0) | 2 (0.5) |
| HPV-18 baseline serostatus, n (%) |  |  |  |
| Seronegative | 462 (91.3) | 355 (93.9) | 356 (88.8) |
| Seropositive | 38 (7.5) | 20 (5.3) | 43 (10.7) |
| Not available | 6 (1.2) | 3 (0.8) | 2 (0.5) |
| 3D_M0,1,6, three-dose schedule administered at months 0, 1 and 6; 2D_M0,6, two-dose schedule administered at months 0 and 6; 2D_M0,12, two-dose schedule administered at months 0 and 12; ATP-I, according-to-protocol immunogenicity cohort; ELISA, enzyme-linked immunosorbent assay; M, month; n (%), number (percentage) of participants in given category; N, number of participants in the cohort; SD, standard deviation.  Seronegative status defined as an antibody titer lower than the assay cut-off prior to vaccination (19 EU/mL for HPV-16 and 18 EU/mL for HPV-18). | | | |
